# Ticagrelor or prasugrel in patients with acute coronary syndrome with off-hour versus on-hour presentation: a subgroup analysis of the ISAR-REACT 5 trial

**DOI:** 10.1007/s00392-022-02040-z

**Published:** 2022-07-05

**Authors:** Michael Behnes, Shqipdona Lahu, Gjin Ndrepepa, Maurizio Menichelli, Katharina Mayer, Jochen Wöhrle, Isabell Bernlochner, Senta Gewalt, Bernhard Witzenbichler, Willibald Hochholzer, Dirk Sibbing, Salvatore Cassese, Dominick J. Angiolillo, Rayyan Hemetsberger, Christian Valina, Arne Müller, Sebastian Kufner, Christian W. Hamm, Erion Xhepa, Alexander Hapfelmeier, Hendrik B. Sager, Michael Joner, Massimiliano Fusaro, Gert Richardt, Karl-Ludwig Laugwitz, Franz-Josef Neumann, Heribert Schunkert, Stefanie Schüpke, Adnan Kastrati, Ibrahim Akin

**Affiliations:** 1 First Department of Medicine, Faculty of Medicine Mannheim, University of Heidelberg, Mannheim, Germany; 2 Department of Cardiology, Deutsches Herzzentrum München, Technische Universität München, Munich, Germany; 3 German Center for Cardiovascular Research (DZHK), Partner Site Munich Heart Alliance, Munich, Germany; 4 Ospedale Fabrizio Spaziani, Cardiology, Frosinone, Italy; 5 Department of Cardiology, Medical Campus Lake Constance, Friedrichshafen, Germany; 6 Medizinische Klinik und Poliklinik Innere Medizin I (Kardiologie, Angiologie, Pneumologie), Klinikum rechts der Isar, Technische Universität München, Munich, Germany; 7 Cardiology and Pneumology, Helios Amper-Klinikum Dachau, Dachau, Germany; 8 Department of Cardiology and Angiology II, University Heart Center Freiburg Bad Krozingen, Bad Krozingen, Germany; 9 Klinikum der Universität München, Ludwig-Maximilians-University, Cardiology, Munich, Germany; 10 Division of Cardiology, University of Florida College of Medicine, Jacksonville, FL USA; 11 Heart Center Bad Segeberg, Bad Segeberg, Germany; 12 Heart Center, Campus Kerckhoff of Justus-Liebig-University, Giessen, Germany; 13 School of Medicine, Institute of AI and Informatics in Medicine, Technical University of Munich, Munich, Germany; 14 School of Medicine, Institute of General Practice and Health Services Research, Technical University of Munich, Munich, Germany

**Keywords:** Acute coronary syndromes, Off-hour presentation, Percutaneous coronary intervention, Prasugrel, Ticagrelor

## Abstract

**Objectives:**

To assess the efficacy and safety of ticagrelor versus prasugrel in patients with acute coronary syndrome (ACS) presenting during off- and on-hours.

**Background:**

The efficacy and safety of ticagrelor versus prasugrel in patients with ACS according to time of hospital presentation remain unknown.

**Methods:**

This post hoc analysis of the ISAR-REACT 5 trial included 1565 patients with ACS presenting off-hours and 2453 patients presenting on-hours, randomized to ticagrelor or prasugrel. The primary endpoint was a composite of death, myocardial infarction, or stroke; the safety endpoint was Bleeding Academic Research Consortium (BARC) type 3–5 bleeding, both at 12 months.

**Results:**

The primary endpoint occurred in 80 patients (10.4%) in the ticagrelor group and 57 patients (7.3%) in the prasugrel group in patients presenting off-hours (hazard ratio [HR] = 1.45; 95% confidence interval [CI] 1.03–2.03; *P* = 0.033), and 104 patients (8.5%) in the ticagrelor group and 80 patients (6.7%) in the prasugrel group in patients presenting on-hours (HR = 1.29 [0.97–1.73]; *P* = 0.085), without significant treatment arm-by-presentation time interaction (P_int_ = 0.62). BARC type 3 to 5 bleeding occurred in 35 patients (5.1%) in the ticagrelor group and 37 patients (5.3%) in the prasugrel group (*P* = 0.84) in patients presenting off-hours, and 60 patients (5.9%) in the ticagrelor group and 43 patients (4.6%) in the prasugrel group in patients presenting on-hours (*P* = 0.17).

**Conclusions:**

In patients with ACS planned to undergo an invasive treatment strategy, time of presentation (off-hours vs. on-hours) does not interact significantly with the relative efficacy and safety of ticagrelor vs. prasugrel.

**Clinical trial registration.:**

NCT01944800.

**Graphical abstract:**

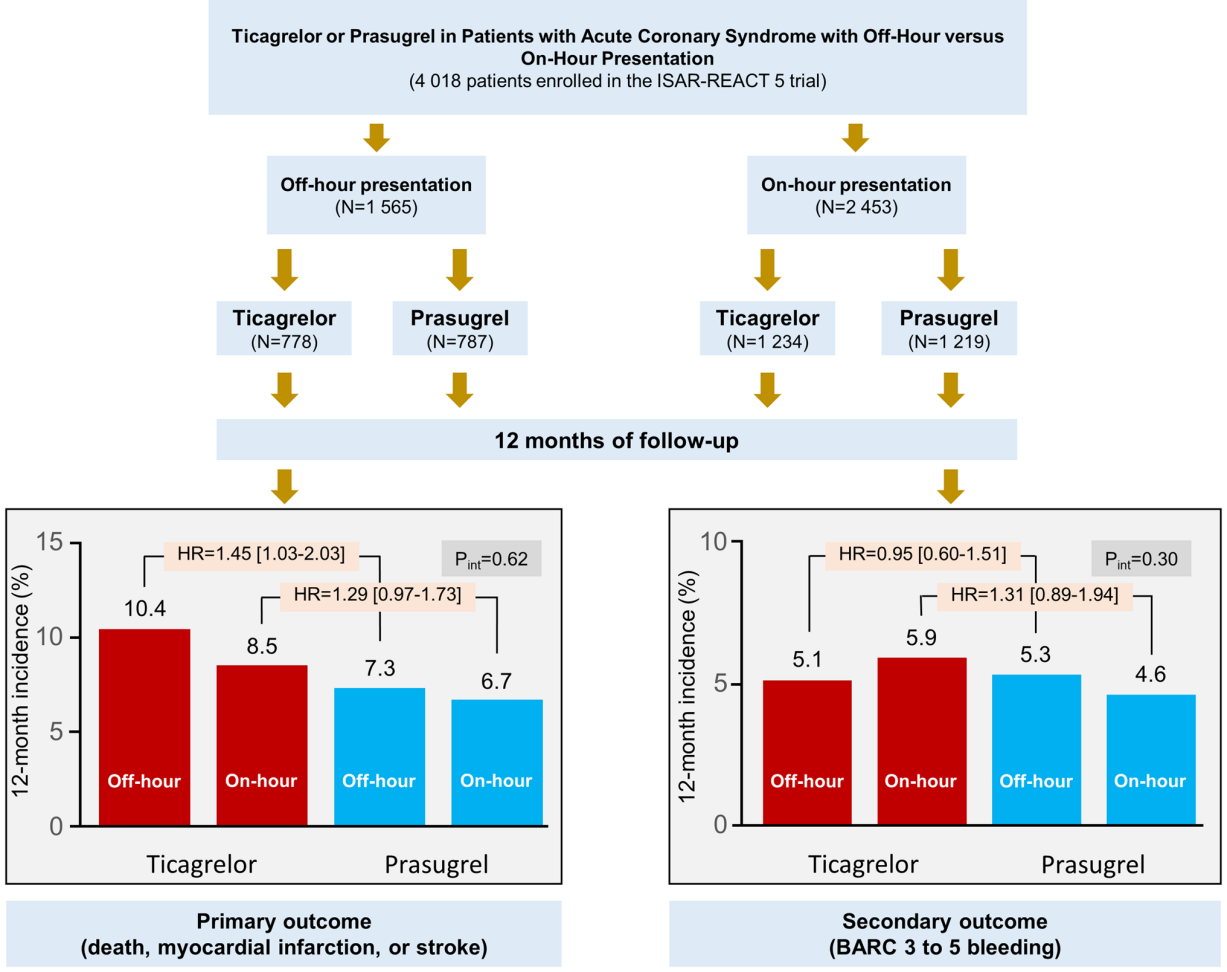

## Introduction

Several studies have reported higher in-hospital and long-term mortality in patients with acute myocardial infarction (MI) presenting during off-hours [1–3]. A shortage of specialized staff [4], lower use of cardiac invasive procedures [1], and longer door-to-balloon times [5, 6] have been reported during off-hour admission, suggesting a worse quality of care in patients presenting to the hospital during off-hours and stressing the need for powerful antiplatelet drugs in these patients. Prasugrel and ticagrelor—the newer P2Y_12_ inhibitors—provide more potent and consistent platelet inhibition compared with clopidogrel and randomized clinical trials have demonstrated an advantage of these drugs over clopidogrel and of prasugrel over ticagrelor in reducing the ischemic risk in patients with acute coronary syndrome (ACS) undergoing percutaneous coronary intervention (PCI) [7–9]. Patients presenting with acute MI during off-hours have a worse cardiovascular risk profile [3, 10, 11] and these patients may especially benefit from potent platelet inhibition with prasugrel [9]. On the other hand, the standard twice daily administration of ticagrelor might be more advantageous compared with the once daily administration of prasugrel in overcoming the periods of increased platelet reactivity and aggregation during the early morning hours [12–14]. Evidence suggests that while platelet inhibition with prasugrel in patients with ACS does not follow a circadian pattern [13], platelet inhibition with ticagrelor might be subject to circadian variations in healthy subjects [15]. In this regard, it is largely unexplored whether the time of day at hospital presentation affects the efficacy and safety of ticagrelor versus prasugrel in patients with ACS managed with an invasive treatment strategy. We undertook this study to investigate whether there are differences in 1-year clinical outcomes between ACS patients treated with ticagrelor and prasugrel planned to undergo an invasive treatment strategy, who presented during off-hours and on-hours.

## Materials and methods

### Patients

This study assessed the efficacy and safety of ticagrelor versus prasugrel in patients with ACS according to off-hour versus on-hour presentation to hospital. The study is a post hoc analysis of the Intracoronary Stenting and Antithrombotic Regimen: Rapid Early Action for Coronary Treatment (ISAR-REACT 5) trial (Clinical Trial Registration: NCT01944800) [9]. The inclusion and exclusion criteria are reported in the primary publication [9]. In brief, patients hospitalized for ACS (unstable angina, non-ST-segment elevation myocardial infarction [NSTEMI], and ST-segment elevation myocardial infarction [STEMI]) planned to undergo an invasive management strategy were included. Patients were randomized to receive ticagrelor (a loading dose of 180 mg as soon as possible after randomization and a maintenance dose of 90 mg twice daily) or prasugrel (a loading dose of 60 mg and a maintenance dose of 10 mg once daily). In patients with NSTE-ACS, the loading dose of prasugrel was given after coronary anatomy was known (i.e., with no pre-treatment before diagnostic coronary angiography) and before proceeding to PCI. In patients with STEMI, prasugrel was given as soon as possible after randomization. In patients ≥ 75 years of age or those with a body weight < 60 kg (irrespective of age), a reduced maintenance dose of prasugrel (5 mg) was recommended [16]. Aspirin therapy included a loading dose of 150–300 mg intravenous or chewed aspirin and a maintenance dose of 75–100 mg daily in both ticagrelor and prasugrel arms. Dual antiplatelet therapy was recommended for at least 1 year. The study protocol was approved by the local ethics committee at each participating center. The study conformed to the Declaration of Helsinki.

### Definitions and outcomes

Regular hours (on-hours) were defined as weekdays (Monday to Friday) from 8 AM to 5 PM. Off-hours were defined as night shift hours (from > 5 PM to < 8 AM), weekends, and local holidays [17]. Differences between the recruitment centers in defining on-hours and off-hours periods were also considered. Based on the time of presentation to hospital, patients were categorized in two groups: those presenting off-hours (*n* = 1565) and those presenting on-hours (*n* = 2453).

The primary (efficacy) endpoint was a composite of death, myocardial infarction, or stroke at 12 months after randomization. The safety endpoint was the incidence of bleeding types 3–5 according to the Bleeding Academic Research Consortium (BARC) at 12 months after randomization. Other endpoints analyzed were the individual components of the primary endpoint, the incidence of cardiovascular death, and stent thrombosis (definite or probable) at 12 months after randomization. Detailed definitions of the study endpoints are provided in the primary publication [9].

### Follow-up

Clinical follow-up was scheduled at 1 month, 6 months, and 1 year after randomization. Patients were contacted by telephone, hospital or outpatient visit, or structured follow-up letter. In case of potential endpoint-related adverse events, source data were solicited. All serious adverse events and efficacy and safety endpoints were monitored on site. In addition, 100% of source data were checked in at least 10% of patients at all centers.

### Statistical analysis

The present analysis was not pre-specified in the study protocol; therefore, it represents a post hoc analysis of a randomized clinical trial. Continuous data are presented as mean ± SD or median (with 25th–75th percentiles) and were compared using either Student’s *t *test or the nonparametric Wilcoxon rank sum test. Categorical variables are presented as counts and proportions, and were compared using the Chi-squared test. The cumulative incidence of the primary endpoint and all-cause death according to study drug (ticagrelor or prasugrel) in patients arriving off-hours versus those arriving on-hours was calculated using the Kaplan–Meier method and the inter-group comparisons were performed using the Cox proportional hazard model. The participating center and stratification according to clinical presentation (ACS with or without ST-segment elevation) were entered into the Cox proportional hazards model as covariates along with study treatment group. For all endpoints, except the primary endpoint and all-cause death, the cumulative incidence functions were computed to account for competing risk. To estimate the interaction between the treatment arm and the time of presentation with respect to study endpoints, an interaction term was entered into the Cox proportional hazards models. Risk estimates are presented as hazard ratios (HR) with 95% confidence intervals (CI). The efficacy endpoint was analyzed according to the intention-to-treat principle including all patients as initially assigned irrespective of the actual treatment received. The safety endpoint of bleeding was analyzed in a modified intention-to-treat population (including all patients with at least one application of the study drug, with bleeding assessed for up to 7 days after discontinuation of the study drug). Patients were analyzed from randomization until death, withdrawal of consent, or last contact date. Statistical analysis was performed using the R, version 3.6.0 (R Foundation for Statistical Computing, Vienna, Austria). A two-sided *p* value < 0.05 was considered to indicate statistical significance.

## Results

### Baseline data in patients presenting during off-hours and on-hours

Of the 4018 patients with ACS, 1565 patients (39%) presented during off-hours and 2453 patients (61%) presented during on-hours (Supplemental Fig. S1). Baseline characteristics are shown in Supplemental Table S1. Patients presenting off-hours were younger, had a higher proportion of smokers, and were more likely to present with STEMI and develop cardiogenic shock. Patients presenting on-hours had a higher proportion of patients with diabetes (including those on insulin therapy), arterial hypertension, hypercholesterolemia, prior PCI, and prior coronary artery bypass grafting (CABG). They had more often a body weight < 60 kg, presented more often with unstable angina and NSTEMI, and were more likely to undergo conservative treatment or CABG than patients presenting off-hours.

Diagnostic coronary angiography was performed in 4004 patients (99.7%). Patients presenting off-hours were more likely to have vascular access via femoral artery, had a higher proportion of patients with single vessel disease, and a lower left-ventricular ejection fraction than patients presenting on-hours (Supplemental Table S2). Patients presenting off-hours had more often TIMI flow grades of 0, 1, and 2 before intervention, and a longer mean total stented length (31.9 mm vs. 29.6 mm; *P* < 0.001). Patients presenting on-hours were more likely to have received peri-procedural unfractionated heparin (Supplemental Table S3). Therapy at discharge is shown in Supplemental Table S4. Patients presenting during off-hours were more likely to be discharged on aspirin, prasugrel, and statin therapy than patients presenting during on-hours.

### Baseline data according to study drugs in patients presenting during off-hours and on-hours

In the off-hour group, 778 patients were assigned to ticagrelor and 787 patients to prasugrel. In the on-hour group, 1234 patients were assigned to ticagrelor and 1219 patients to prasugrel. Baseline data are shown in Table 1. In the off-hour group, baseline characteristics did not differ significantly according to study drug (ticagrelor or prasugrel), with the exception of the proportions of patients with active smoking and cardiogenic shock (higher proportions of prasugrel-assigned patients had these conditions). In the on-hour group, baseline characteristics were well-balanced, with no statistically significant differences according to study drug.

**Table 1:** Baseline data according to study drug in patients presenting during off-hours and on-hours

Characteristic	Off-hours (*N* = 1565)			On-hours (*N* = 2453)		
	Ticagrelor (*N* = 778)	Prasugrel (*N* = 787)	*P* value	Ticagrelor (*N* = 1234)	Prasugrel (*N* = 1219)	*P* value
Age, mean (SD), year	63.1 ± 12.4	63.1 ± 12.0	> 0.99	65.4 ± 11.7	65.6 ± 12.0	0.61
Sex			0.76			0.75
Female—no. (%)	177 (22.8)	173 (22.0)		301 (24.4)	305 (25.0)	
Male—no. (%)	601 (77.2)	614 (78.0)		933 (75.6)	914 (75.0)	
Diabetes—no. (%)	169/777 (21.8)	145 (18.4)	0.11	294 (23.8)	284/1218 (23.3)	0.80
Insulin-treated—no. (%)	45/777 (5.8)	42 (5.3)	0.78	98 (7.9)	95/1218 (7.8)	0.96
Smoking—no. (%)	284/774 (36.7)	307/784 (39.2)	0.047	398/1228 (32.4)	360/1215 (29.6)	0.30
Arterial hypertension—no. (%)	507/776 (65.3)	496/786 (63.1)	0.39	925/1232 (75.1)	888/1217 (73.0)	0.25
Hypercholesterolemia—no (%)	415/776 (53.5)	427 (54.3)	0.80	763/1231 (62.0)	736/1216 (60.5)	0.49
Prior myocardial infarction—no. (%)	110/777 (14.2)	132 (16.8)	0.17	201/1233 (16.3)	188/1218 (15.4)	0.59
Prior PCI—no. (%)	155/777 (19.9)	156/786 (19.8)	> 0.99	298 (24.1)	307/1218 (25.2)	0.58
Prior CABG—no. (%)	35/777 (4.5)	41/786 (5.2)	0.59	80 (6.5)	89 (7.3)	0.47
Cardiogenic shock—no. (%)	11 (1.4)	24 (3.1)	0.044	20 (1.6)	10 (0.8)	0.11
Systolic blood pressure, mean (SD), mmHg	142 ± 25.0	141 ± 25.6	0.41	144 ± 25.0	144 (23.7)	0.64
Diastolic blood pressure, mean (SD), mmHg	82.5 ± 14.4	81.8 ± 14.3	0.37	81.7 ± 14.7	81.8 (13.5)	0.89
Heart rate, mean (SD), beats/min	78.4 ± 16.5	76.9 ± 15.9	0.062	76.0 ± 15.5	75.5 ± 15.3	0.36
Body mass index, mean (SD), kg/m^2^	27.9 ± 4.6	28.0 ± 4.5	0.75	27.7 ± 4.7	27.7 ± 4.4	0.98
Weight < 60 kg—no. (%)	33/770 (4.3)	30/779 (3.9)	0.76	75/1233 (6.1)	64 (5.3)	0.45
Creatinine, mean (SD), µmol/L	88.5 ± 26.8	89.9 ± 34.4	0.40	87.2 ± 27.7	87.1 ± 27.6	0.96
Diagnosis at admission			0.37			0.51
Unstable angina—no. (%)	46 (5.9)	56 (7.1)		203 (16.5)	205 (16.8)	
NSTEMI—no. (%)	319 (41.0)	299 (38.0)		611 (49.5)	626 (51.4)	
STEMI—no. (%)	413 (53.1)	432 (54.9)		420 (34.0)	388 (31.8)	
Coronary angiography—no. (%)	777 (99.9)	784 (99.6)	0.63	1226 (99.4)	1217 (99.8)	0.11
Treatment strategy—no. (%)			0.042			0.75
PCI	676 (86.9)	714 (90.8)		1000 (81.3)	987 (81.0)	
CABG	15 (1.9)	9 (1.2)		32 (2.6)	27 (2.2)	
Conservative	87 (11.2)	63 (8.0)		198 (16.1)	205 (16.8)	

Missing continuous data: Off-hours group: systolic blood pressure, 2 patients in the prasugrel group; diastolic blood pressure, 11 patients (7 in the prasugrel group, 4 in the ticagrelor group); heart rate, 1 patient in the prasugrel group; body mass index, 18 patients (9 in each group). On-hours group: systolic blood pressure, 1 patient in the ticagrelor group; diastolic blood pressure, 5 patients (3 patients in the ticagrelor group, 2 patients in the prasugrel group); heart rate, 1 patient in the ticagrelor group; body mass index, 13 patients (3 in the ticagrelor group, 10 in the prasugrel group); the remaining continuous data were complete

*CABG* coronary artery bypass grafting, *NSTEMI* non-ST-segment elevation myocardial infarction, *PCI* percutaneous coronary intervention, *STEMI* ST-segment elevation myocardial infarction

Angiographic (Supplemental Table S5), procedural (Supplemental Table S6) data, and drug therapy at discharge (Supplemental Table S7) appear to differ little between ticagrelor- and prasugrel-assigned patients presenting during off-hours and on-hours.

### Clinical outcomes

The follow-up was complete in all but 90 patients (2.2%): 32 patients presenting during off-hours and 58 patients presenting during on-hours (2.0% vs. 2.4%, respectively; *P* = 0.50). The primary endpoint (death, myocardial infarction, or stroke at 1 year after randomization) occurred in 137 patients presenting off-hours and 184 patients presenting on-hours (cumulative incidence 8.9% vs. 7.6%, respectively; hazard ratio [HR] = 1.18, 95% confidence interval [CI] 0.94–1.47; *P* = 0.15; Supplemental Fig. S2, left panel). The probable and definite stent thrombosis (1.7% vs. 0.8%; *P* = 0.015), and definite stent thrombosis (1.2% vs. 0.6%; *P* = 0.044) were more frequent in patients presenting off-hours than those presenting on-hours. Clinical outcomes according to presentation during off-hours and on-hours are shown in Supplemental Table S8.

**Fig. 1:**
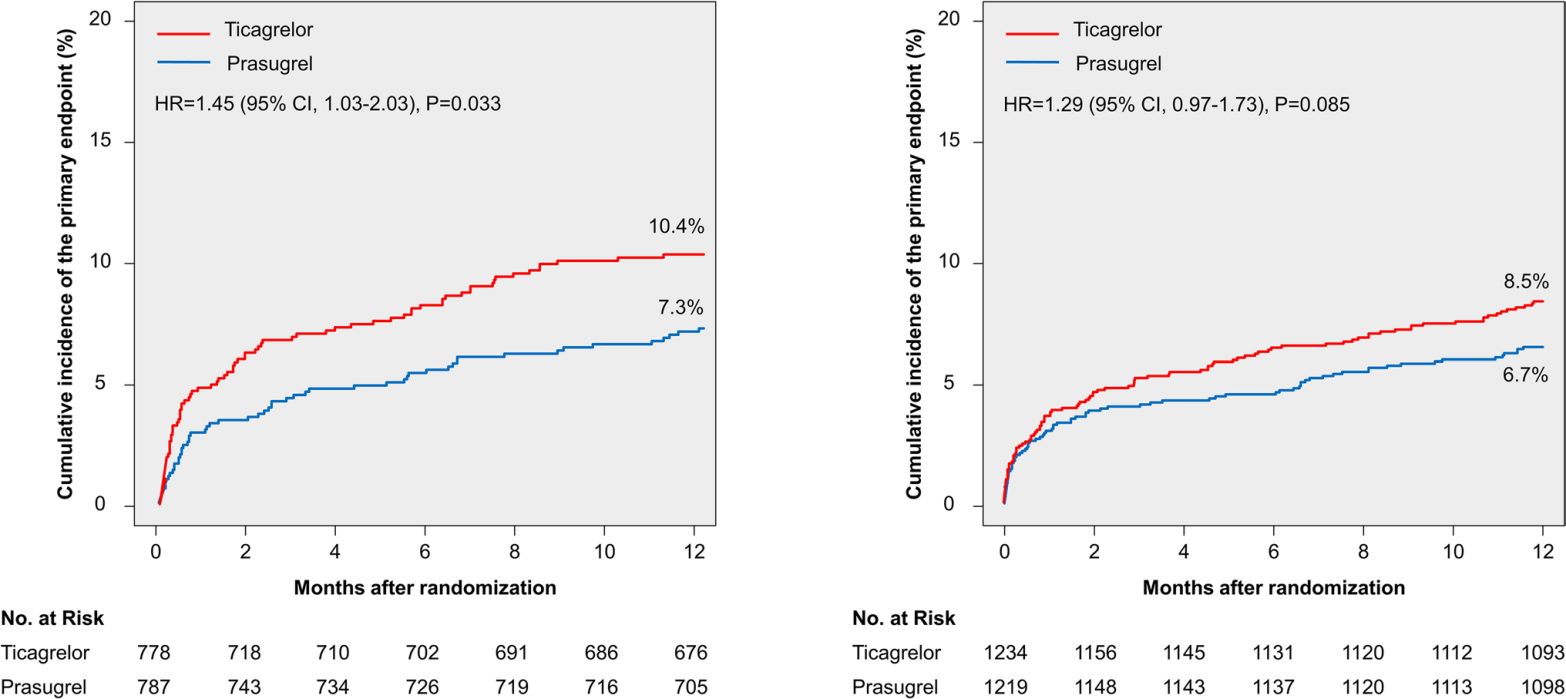
One-year cumulative incidence of the primary endpoint (death, myocardial infarction, or stroke). Left panel: the incidence of the primary endpoint in patients presenting during off-hours. Right panel: the incidence of the primary endpoint in patients presenting during on-hours. Primary endpoint was evaluated in the intention-to-treat population *CI *confidence interval, *HR*  hazard ratio

Clinical outcomes according to study drug are shown in Table 2. In patients presenting off-hours, the primary endpoint occurred in 80 patients in the ticagrelor group and 57 patients in the prasugrel group (cumulative incidence 10.4% and 7.3%, respectively; HR = 1.45 [1.03–2.03]; *P* = 0.033; (Fig. 1, left panel). In patients presenting on-hours, the primary endpoint occurred in 104 patients in the ticagrelor group and 80 patients in the prasugrel group (cumulative incidence 8.5% and 6.7%, respectively; HR = 1.29 [0.97–1.73]; *P* = 0.085; Fig. 1, right panel). There was no significant treatment arm-by-presentation time interaction with respect to primary outcome (*P* for interaction = 0.62).

**Table 2:** Clinical outcomes according to study drug in patients presenting during off-hours and on-hours

Outcome	Off-hours (*N* = 1565)				On-hours (*N* = 2453)			
	Ticagrelor (*N* = 778)	Prasugrel (*N* = 787)	HR [95% CI]	*P* value	Ticagrelor (*N* = 1234)	Prasugrel (*N* = 1219)	HR [95% CI]	*P* value
Primary endpoint (death, myocardial infarction, or stroke)	80 (10.4)	57 (7.3)	1.45 [1.03–2.03]	0.033	104 (8.5)	80 (6.7)	1.29 [0.97–1.73]	0.085
Death	36 (4.7)	35 (4.5)	1.04 [0.65–1.66]	0.87	54 (4.4)	38 (3.2)	1.41 [0.93–2.13]	0.11
Cardiovascular	27	32			36	27		
Non-cardiovascular	9	3			18	11		
Myocardial infarction	45 (5.8)	22 (2.8)	2.11 [1.27–3.51]	0.004	51 (4.2)	38 (3.2)	1.34 [0.88–2.03]	0.18
Type 1	21	14			31	21		
Type 2	2	2			2	1		
Type 4a	8	2			11	9		
Type 4b	14	4			6	7		
Type 5	0	0			1	0		
STEMI	14	3			17	11		
Stroke	10 (1.3)	7 (0.9)	1.45 [0.55–3.80]	0.45	12 (1.0)	12 (1.0)	0.99 [0.44–2.20]	0.98
Ischemic	7	5			9	12		
Hemorrhagic	3	2			3	0		
Definite or probable stent thrombosis	17 (2.2)	9 (1.2)	1.93 [0.86–4.32]	0.11	9 (0.7)	11 (0.9)	0.81 [0.33–1.95]	0.64
Definite stent thrombosis	15 (1.9)	4 (0.5)	3.82 [1.27–11.52]	0.017	7 (0.6)	8 (0.7)	0.86 [0.31–2.38]	0.78
BARC type 3–5 bleeding^a^	35/771 (5.1)	37/732 (5.3)	0.95 [0.60–1.51]	0.84	60/1218 (5.9)	43/1041 (4.6)	1.31 [0.89–1.94]	0.17
3a	12	19			35	22		
3b	16	14			16	17		
3c	1	0			3	2		
4	3	2			5	0		
5a	1	0			0	0		
5b	2	2			1	2		

Data are numbers of events with Kaplan–Meier estimates (%) for the primary endpoint and death, as well as cumulative incidence (%) after accounting for competing risk for the remaining endpoints

*BARC* Bleeding Academic Research Consortium, *CI* confidence interval, *HR* hazard ratio, *STEMI* ST-segment elevation myocardial infarction

^a^BARC type 3–5 bleeding was analyzed according to the modified intention-to-treat principle

In patients presenting off-hours, the incidence of definite stent thrombosis was lower in the prasugrel arm than in the ticagrelor arm (0.5% vs. 1.9%; *P* = 0.017). In patients presenting on-hours, there were numerically fewer deaths in the prasugrel arm (3.2% vs. 4.4%; *P* = 0.11) than in patients in the ticagrelor arm (Table 2).

### Bleeding events

**Fig. 2:**
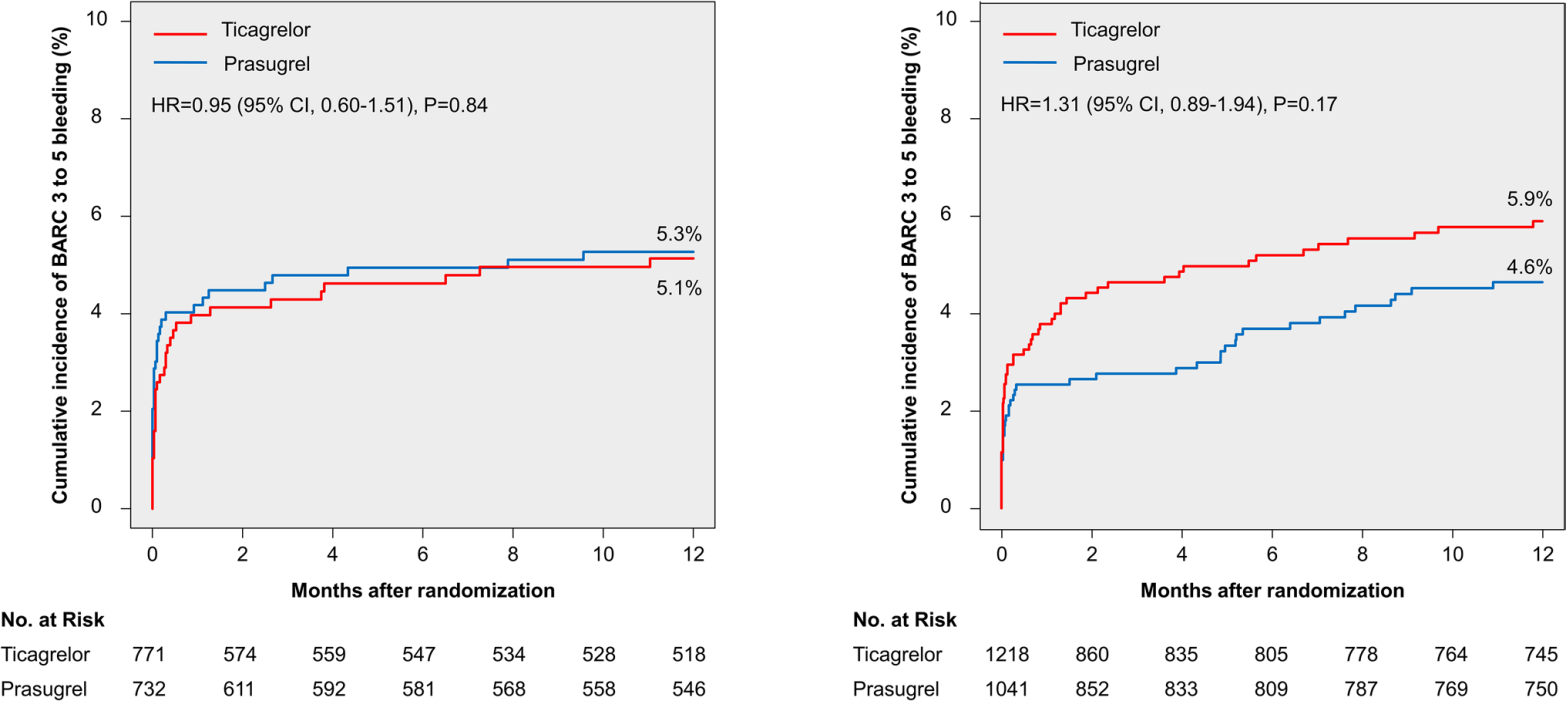
Cumulative incidence of the secondary safety endpoint (1-year incidence of Bleeding Academic Research Consortium type 3–5 bleeding). BARC type 3–5 bleeding was evaluated in the modified intention-to-treat population after accounting for the competing risk of death. Results are presented for patients presenting during off-hours (left panel) and on-hours (right panel). *BARC* Bleeding Academic Research Consortium, *CI *confidence interval, *HR* hazard ratio

Bleeding events according to presenting hours are shown in Supplemental Table S8. BARC 3–5 bleeding occurred in 88 patients presenting off-hours and 138 patients presenting on-hours (cumulative incidence 5.7% and 5.7%; HR = 1.01 [0.77–1.32]; *P* = 0.95; Supplemental Fig. S2, right panel). With regard to study drug, in patients presenting off-hours, BARC 3–5 bleeding occurred in 35 patients in the ticagrelor group and 37 patients in the prasugrel group (cumulative incidence 5.1% vs. 5.3%; HR = 0.95 [0.60–1.51]; *P* = 0.84; Fig. 2, left panel). In patients presenting on-hours, BARC 3–5 bleeding occurred in 60 patients in the ticagrelor group and 43 patients in the prasugrel group (cumulative incidence 5.9% vs. 4.6%; HR = 1.31 [0.89–1.94]; *P* = 0.17; Fig. 2, right panel). There was no treatment arm-by-presentation time interaction regarding the occurrence of BARC type 3 to 5 bleeding (*p* for interaction = 0.30). Individual classes of bleeding according to ticagrelor or prasugrel in patients presenting off-hours and on-hours are shown in Table 2.

## Discussion

In this study, we assessed whether there are differences in the efficacy and safety of ticagrelor versus prasugrel in patients with ACS treated with an invasive treatment strategy, according to off-hour versus on-hour presentation to hospital. The main findings of the study may be summarized as follows: (1) the efficacy of ticagrelor versus prasugrel appears not to differ according to time of hospital arrival; the reduction in the 12-month incidence of ischemic events by prasugrel compared with ticagrelor was consistent among patients presenting during off-and on-hours, albeit with different risk estimates. (2) Therapy with ticagrelor or prasugrel appears to be associated with a similar risk of bleeding regardless of presentation time.

Several studies have investigated the potential impact of arrival times and optimal timing of invasive PCI on outcomes of patients with ACS (STEMI, NSTEMI or both). A subgroup analysis of the Harmonizing Outcomes with Revascularization and Stents in Acute Myocardial Infarction (HORIZONS-AMI) trial showed longer “door-to-balloon” and total ischemic times in STEMI patients presenting during off-hours (50.6%) compared with patients presenting on-hours. However, these longer time intervals did not affect any clinical outcome at follow-up, but their impact on the efficacy of drugs, such as bivalirudin, unfractionated heparin, or GPI, was not investigated [5].

The American Heart Association (AHA) “Get With The Guidelines–Coronary Artery Disease” (GWTG-CAD) registry evaluated the impact of arrival time on the care and outcomes of 93,595 patients with ACS treated in 379 hospitals between July 2000 and September 2005 [10]. In the final analysis cohort (*n* = 62,814 patients; 32.3% with STEMI and 67.7% with NSTEMI), 46% of patients presented during regular hours, and 54% of patients presented during off-hours. Despite slightly lower rates of primary PCI and revascularization and longer door-to-balloon times during the initial hospitalization in patients presenting during off-hours, in-hospital mortality was similar in both patient groups [10]. A 2014 meta-analysis with a total of 1,892,424 patients with ACS from the United States, Canada, and Europe [6], and a subsequent retrospective study [18] demonstrated that patients with ACS presenting during off-hours had higher in-hospital and 30-day mortality than patients presenting during on-hours and this difference was even larger in patients with STEMI [6, 18]. A number of factors that may underlie a worse prognosis in patients with ACS presenting off-hours compared with patients presenting on-hours have been suggested. Thus, walk-in or self-transported patients with ACS [19, 20], absence of digital prehospital ECG transfer [21], insufficient centralized EMS networking [22], lack of patient awareness programs [23], resource constrained hospitals [24], slow initial triage, absence of dedicated in-hospital pathways [25], major complications such as those related to emergency CABG surgery, ventricular tachyarrhythmias, stroke or transient ischemic attack, bleedings from gastrointestinal, retroperitoneal, or intracranial origin [18], higher amount of contrast use and associated contrast-induced nephropathy [26], socioeconomic differences in between the countries and regions [6], fatigue of medical staff and varying expertise of the individual PCI operators, and circadian variation in myocardial perfusion and increased reperfusion times have been suggested.

The ISAR-REACT 5 trial recruited patients predominantly from the urban areas in Germany (18 different cities) and Italy (Florence and Frosinone). In Germany, there is a widespread standardized and well-established emergency network to optimally supply patients with ACS. The dedicated German chest pain unit network has been reported to ensure rapid and structured prehospital and in-hospital care and may compensate for longer door-to-balloon times during off-hour presentation by shortening symptom-to-admission or symptom-to-first medical contact time intervals [23, 25]. Likewise, in Italy, efficient networks to guarantee efficient reperfusion therapies for patients with ACS have shown comparable clinical effectiveness both during off-hours and regular hours [27].

The current study showed that prasugrel was superior to ticagrelor (both on top of aspirin) in terms of prevention of ischemic events at 1 year in patients with ACS, regardless of presentation during off-hours or on-hours. Importantly, there were no significant differences in the risk for bleeding between the drugs in patients presenting off-hours and on-hours. The present study may be the first to demonstrate the beneficial prognostic impact of the guideline-recommended pharmacotherapy in patients with ACS undergoing invasive treatment and presenting off-hours. Thus, our data suggest that prasugrel may outbalance or even overcome the described healthcare system- and staff-related factors associated with a worse prognosis in patients with ACS who present to hospital during off-hours.

Reasons why prasugrel showed a somewhat better efficacy in patients presenting off-hours remain unknown. Pharmacokinetics and pharmacodynamics of P2Y_12_ antagonists and on-treatment platelet reactivity with P2Y_12_ antagonists are potentially influenced by sex, body weight, chronic kidney disease, genetics, smoking, diabetes, body mass, inflammation, and drug–drug interactions [28, 29]. Although clear differences in the cardiovascular risk profile with a differential impact on the efficacy and safety of prasugrel or ticagrelor in patients presenting off-hours or on-hours were not observed, an influence of these factors cannot entirely be excluded. Patients presenting off-hours were more likely to have been treated with PCI. Since prasugrel may be particularly advantageous in protecting from ischemic events in patients with ACS after PCI, this could explain, at least partially, the better efficacy of the drug in patients with ACS presenting off-hours. The finding that prasugrel reduced significantly the incidence of definite stent thrombosis in patients presenting off-hours seems to support this contention.

In addition, adherence to medication may have contributed to the differences in the primary outcome of patients with ACS presenting during off-hours who were assigned to ticagrelor. Non-adherence to cardiovascular (poly-) pharmacotherapies is a common finding [28], and it has recently been described for antihypertensive drugs, angiotensin-converting enzyme inhibitors, statins, and P2Y_12_ inhibitors following index ACS events [29–31]. In the ISAR-REACT 5 trial, the frequency of drug discontinuation was significantly higher for ticagrelor than prasugrel [9]. In the current analysis, prasugrel was more commonly prescribed at discharge in patients presenting off-hours than those presenting on-hours. Thus, poorer adherence to ticagrelor and/or more frequent prescription of prasugrel in patients presenting off-hours may have contributed to differences in definite stent thrombosis between the drugs.

### Limitations

This study is a non-pre-specified analysis of a randomized trial. Thus, categorization of patients in groups according to time of hospital presentation reduces the study power to reliably prove the superior efficacy of prasugrel or ticagrelor in terms of reduction of ischemic events according to presentation during off-hours or on-hours. In this regard, current findings should be seen as exploratory or hypothesis-generating. Furthermore, the differences in timing of loading of prasugrel (after diagnostic coronary angiography in patients with NSTEMI and unstable angina) may have influenced the risk for bleeding [32]. Finally, this study does not provide mechanistic information as to whether there are differences in pharmacokinetic profiles of prasugrel or ticagrelor related to differences in the baseline risk or ischemia time intervals in patients with ACS presenting off-hours or on-hours.

## Conclusions

In patients with ACS planned to undergo an invasive treatment strategy, time of presentation (off-hours vs. on-hours) does not interact significantly with the relative efficacy and safety of ticagrelor vs. prasugrel. The reduction in the 12-month incidence of ischemic events by prasugrel compared with ticagrelor was consistent regardless of presentation during off-hours or on-hours, albeit with different risk estimates. The risk for bleeding appears to be similar between ticagrelor and prasugrel, regardless of presentation time.
